# Roughhousing with Ions: Surface-Induced Dissociation and Electron Capture Dissociation as Diagnostics of Q-Cyclic IMS-TOF Instrument Tuning Gentleness

**DOI:** 10.1021/jasms.4c00417

**Published:** 2024-12-07

**Authors:** Andrew J. Arslanian, Vicki H. Wysocki

**Affiliations:** 1 Native MS Guided Structural Biology Center, The Ohio State University, Columbus, Ohio, 43210, United States; 2 Department of Chemistry and Biochemistry, The Ohio State University, Columbus, Ohio, 43210, United States

## Abstract

Native mass spectrometry can characterize a range of biomolecular features pertinent to structural biology, including intact mass, stoichiometry, ligand-bound states, and topology. However, when an instrument’s ionization source is tuned to maximize signal intensity or adduct removal, it is possible that the biomolecular complex’s tertiary and quaternary structures can be rearranged in a way that no longer reflect its native-like conformation. This could affect downstream ion activation experiments, leading to erroneous conclusions about the native-like structure. One such activation strategy is surface-induced dissociation (SID), which generally causes native-like protein complexes to dissociate along the weakest subunit interfaces, revealing critical information about the complex’s native-like topology and subunit connectivity. If the quaternary structure has been disturbed then the SID fingerprint will shift as well. Thus, SID was used diagnose source-induced quaternary structure rearrangement and help tune an instrument’s source and other upstream transmission regions to strike the balance between signal intensity, adduct removal, and conserving the native-like structure. Complementary to SID, electron-capture dissociation (ECD) can also diagnose rearranged quaternary structures and was used after in-source activation to confirm that the subunit interfaces were rearranged, opening the structure to electron capture and subsequent dissociation. These results provide a valuable guide for new practitioners to native mass spectrometry and highlight the importance of using standard protein complexes when tuning new instrument platforms for optimal native mass spectrometry performance.

## Graphical Abstract

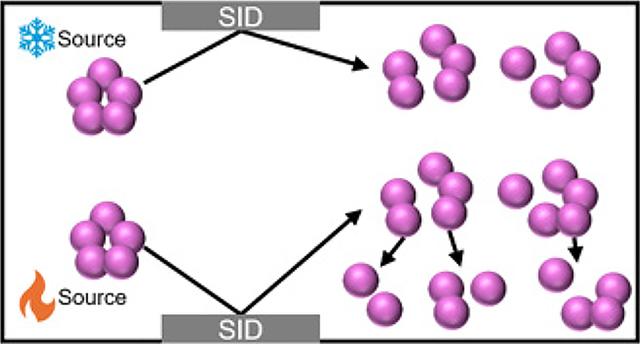

The TOC graphic portrays two distinct SID pathways which depend on either a cold (snowflake) or hot (flame) source. The cold source results in native-like SID for the portrayed homopentameric protein complex, while the hot source results in post-SID secondary fragmentation due to excess energy deposition during in-source activation.

## INTRODUCTION

In native mass spectrometry (nMS) for structural biology, in-source collisional activation is often used to clean up biomolecules and complexes that are adducted by salt and water molecules. This strategy can result in ‘cleaner’ biomolecules and narrower peaks that increase spectral resolution.^1–4^ These two outcomes result in deconvolved masses that are accurate, and easier to obtain than when the complex is ‘adducted,’ leading to broad peaks. In-source collisional activation (isCA) can also be used as a pseudo-MS^n^ step to dissociate the precursor macromolecular complex, or dislodge native ligands, for targeted experiments downstream from the source.^5^ However, it is also known that isCA can cause native-like biomolecular complexes to restructure such that downstream activation steps and analyses do not reflect the native-like structure.^6–12^ Hence, it is critically important to balance the need for ‘cleaner’ mass spectra with the need to produce ionized biological macromolecular complexes that produce data reflecting the native-like structure and chemistry.^13^ However, it can be difficult to ascertain what degree of isCA is appropriate, while also resisting the natural urge to obtain spectra that look ‘nice’, i.e., spectra with narrow, resolved peaks.^14^ This paper seeks to illustrate why care must be taken with isCA when pursuing nMS, by using surface-induced dissociation and electron capture dissociation as tools to develop appropriate “native” instrument tuning. In particular, this paper assesses native-like tuning for a cyclic IMS QTOF instrument with a commercial implementation of SID installed after the selection quadrupole.

Surface-induced dissociation (SID) causes native-like protein complexes to dissociate along the weakest subunit interfaces, providing information about subunit stoichiometry and connectivity, assuming that no intertwined subunits exist and/or that restructuring is not a necessary path to dissociation.^15–19^ However, when isCA causes rearrangement to a non-native-like structure, the protein complexes will no longer produce native-like SID fragmentation patterns.^6,7,20–22^ By this we mean that protein complexes will not dissociate through pathways that indicate their native-like topology, subunit connectivity, etc.^7^ Work published by the Wysocki lab in 2015 illustrates this point.^6^ As protein complexes were subjected to various degrees of isCA, they underwent structural rearrangement that was reflected in the SID spectrum. Collision-induced dissociation (CID) was not able to detect these structural rearrangements because the CID products are typically a highly charged monomer and the complementary (N-1mer) subunit, which does not possess a native-like topology.^15,23^ Thus, SID proved to be a useful probe of source-induced structural rearrangements and can be used to tune isCA to maintain native-like structures.

Electron capture dissociation (ECD) is an ion activation strategy that involves ions interacting with low-energy electrons to induce fragmentation. For proteins, the electron capture process causes the N-C_α_ bond to fragment, producing c/z-type fragments, in comparison to the b/y-type fragments typically obtained by CID.^24,25^ Because these fragments generally come from flexible and solvent-exposed regions, the hydrophobic core is often left intact after the ECD process. This can provide information about the gas phase structures of native-like protein complexes.^26^ Historically, this technique was limited to FTICR instruments due to the feasibility of trapping electrons in the ICR cell to interact with trapped protein cations. When combined with long trapping times, FTICR-based ECD experiments on small proteins revealed that, on long time scales, proteins can elongate and collapse in ways that affect ECD fragmentation.^27–29^ While those studies took place in an FTICR instrument, the Ruotolo lab recently illustrated that ECD could also be sensitive to source-induced structural changes on an IM-Q-TOF instrument.^30^

The work presented here examines a recently developed cyclic IMS QTOF platform that features commercially available SID and ExD devices.^31–33^ We demonstrate which pre-IM regions can be prone to activating native-like proteins and protein complexes, and make recommendations on how to tune those regions. We also demonstrate how isCA can result in quaternary structure rearrangement and how SID can be a sensitive diagnostic tool of that effect. While isCA – **fixed** SID experiments were performed by the Wysocki lab in the past,^6,7,20,21^ those experiments demonstrated the effect of isCA on SID through large jumps between isCA energies. For the first time, this work demonstrates the same effect, in greater detail, by varying isCA in small steps while tracking the effect on the SID pathways for standard protein complexes, while also reporting protein complex drift time changes and monomer charge states, during the isCA – **fixed** SID experiments. Overall, we illustrate that SID, in particular, and ECD, can serve as independent means to validate an instrument’s source tuning to strike the balance between maximum signal intensity and adduct removal, while maintaining native-like structure for protein complexes.

## EXPERIMENTAL SECTION

Ubiquitin from bovine erythrocytes was purchased from Sigma Aldrich (Ubq, monomer, 8.5 kDa, P/N U6253), streptavidin (SA, tetramer, 53 kDa) was purchased from Thermo Scientific (catalog number 21125), human recombinant C-reactive protein (CRP, pentamer, 115 kDa) was purchased from EMD Millipore (P/N 236608), and phosphorylase B (PhosB, dimer, 197 kDa) was purchased from Millipore Sigma (P/N P6635). Tryptophan RNA-binding attenuation protein (TRAP) from *Bacillus stearothermophilus* (*bst*) was obtained from Prof. Mark Foster’s lab at The Ohio State University. It was expressed and purified as previously described, with the minor alteration that cell lysing was accomplished via sonication after overnight expression.^34^ Proteins purchased as lyophilized powder were reconstituted in 18 MΩ water to a protein complex concentration of ~40 μM, except for Ubq, which was reconstituted to 100 μM. All proteins were buffer exchanged twice into 200 mM ammonium acetate using BioRad Microspin 6 buffer exchange columns and diluted to 1 μM for CRP, 2 – 3 μM for SA, and 4 uM for PhosB in 160/40 ammonium acetate/triethylammonium acetate, except for Ubq which was diluted to 10 μM with 200 mM ammonium acetate. TRAP was provided at a protein complex concentration of ~1.5 μM in 600 mM ammonium acetate. The 21+ charge state was obtained by spraying from this stock solution. The 15+ charge state was obtained by adding 1 μL of TEAA to 4 μL of the stock solution. For all complexes, the triethylammonium acetate (Sigma Aldrich, P/N 90358) was added for chemical charge reduction because lower charge states often give native-like fragmentation patterns compared to normal charge states from 200 mM ammonium acetate.^35–42^

Ions were introduced into the instrument via nanoelectrospray ionization, using glass capillaries (Sutter Instruments) pulled on a Sutter Instruments P-97 tip puller (Novato, CA). Electrospray voltages were ramped up to 1.3 kV to induce spray, then were ramped down to 0.7 – 1.0 kV to sustain spray.

All experiments were performed on a Waters SELECT Series Cyclic IMS (Q-IM-TOF) with a commercially available, stainless steel, thin SID device (3-mm along the ion path) located after the selection quadrupole in place of the DRE lens prior to the Trap stacked ring ion guide used for CID.^31^ The instrument was operated in ion mobility mode, with ion mobility separation traveling wave (TW) static height of 25 V and TW velocity of 375 m/s. All instrument settings were tuned to be as gentle as possible to minimize protein complex restructuring, unless collision-induced structural changes were intended. In that case, the cone voltage was adjusted in 10 V steps from 0 – 160 V to vary the amount of isCA (named “cone isCA” by Waters for consistency with older instruments, although the voltage variation does not involve a source cone). Subsequently, data were acquired without further activation (cone isCA), or with additional, intentional activation by another method (cone isCA – **fixed** SID or cone isCA – ECD). Data acquired via cone isCA were collected in triplicate on three separate days. Data processing was accomplished using CIUSuite 2.^43^

Cone isCA – **fixed** SID experiments were performed at a **fixed** SID potential, for the indicated quadrupole-selected charge states, while **scanning the cone voltage** in 10 V steps. All data were obtained in triplicate. Final plots are the subsequent averages and error bars represent one standard deviation from the mean. For SA 11+ and CRP 17+, the SID potential was 45 V (495 and 765 eV, respectively). For PhosB 21+ the SID potential was 190 V (3990 eV). For TRAP the SID potential was 70 V for the 15+ charge state (1050 eV) and 50 V for the 21+ charge state (1050 eV). SID data for SA, CRP, and TRAP were processed using DriftScope v3.0 to extract oligomer intensities to be sum normalized and plotted as a function of cone voltage. The same procedure was followed to obtain weighted average SID monomer charge plots. When the SID data for PhosB were visualized in DriftScope, the precursor dimer and product monomer lines overlapped in a way that prevented signal intensity extraction. Thus, an alternative strategy was devised. First, Waters Raw files were converted to mzML files using ProteoWizard MSconvert^44^ (version 3.0.23244-bc8a3ad) with the Scan Summing filter applied with the following parameters: Precursor tolerance ±0.05 m/z, Scan time tolerance ±5 s, ion mobility tolerance ±5 ms, and Sum MS1 scans. The mzML files were then imported to an HDF file and deconvolved using MetaUniDec,^45,46^ with the cone voltage as the input variable. MetaUniDec was then used to extract signal intensities and charge states for PhosB’s monomer and dimer at each cone voltage.

Cone isCA – ECD experiments were conducted at two different cone settings, either 20 or 160 V, followed by quadrupole isolation of the same charge states as were selected for the isCA – SID experiments, then by IM separation, and finally by electron capture in the ExD device located in the pre-transfer guide after IM.^32^ Because ECD product ions tend to remain “stuck” (non-covalently bound) within native-like proteins/complexes due to non-covalent interactions,^26^ post-ECD activation (transfer collision energy (TCE)) was used after electron capture to liberate the fragments. Typical Transfer CE voltages ranged from 60 – 130 V. All ECD spectra were processed using ExDViewer v4.5.8 (eMSion, an Agilent company), with the default deconvolution and peak picking settings and a mass tolerance of 20 ppm for product ion matching.^47^

## RESULTS AND DISCUSSION

### Experiment Type: Optimize Pre-IM Tuning

**Figure 1.**
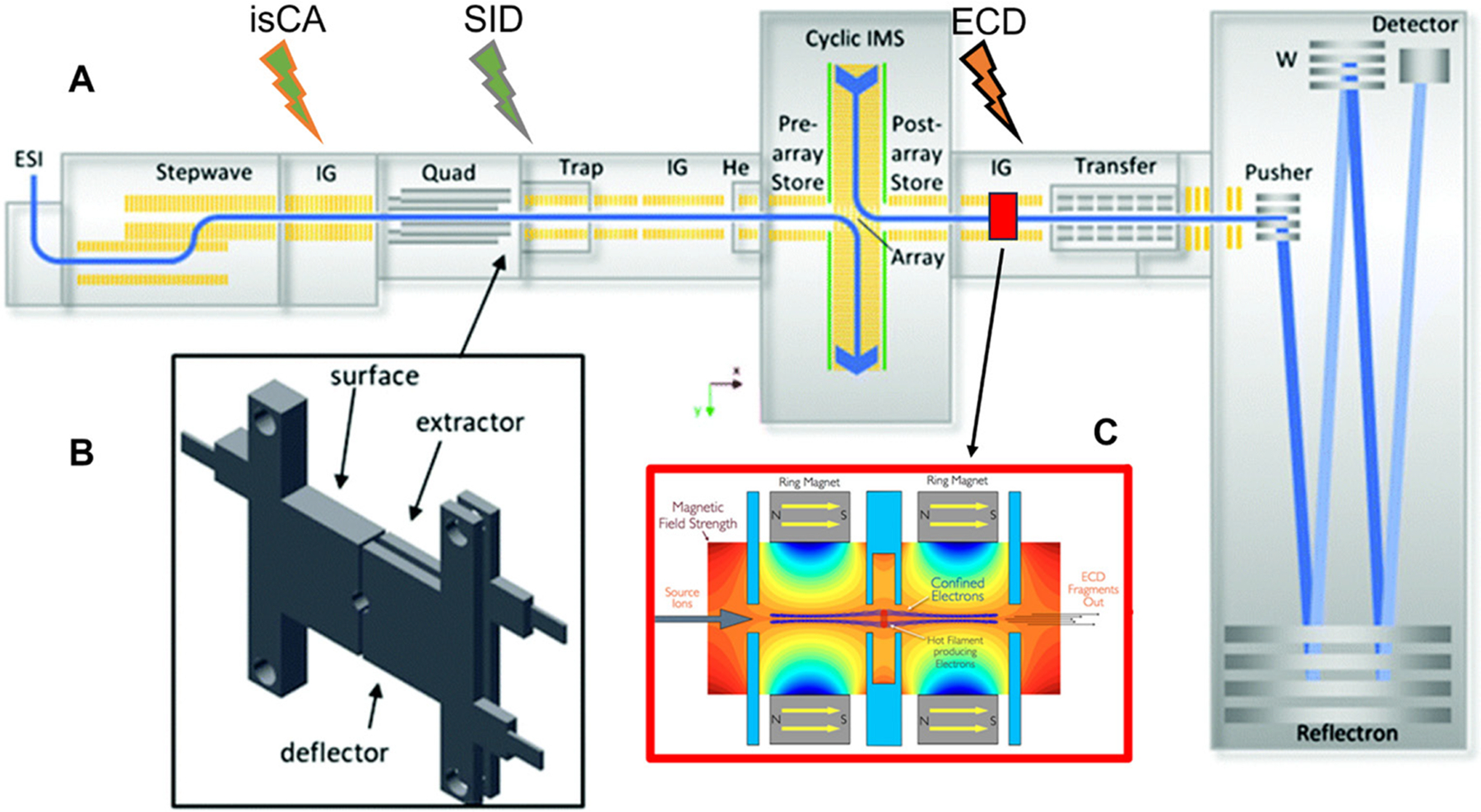
Instrument diagram for the Waters SELECT Series Cyclic IMS (Q-IM-TOF). A) The instrument itself, complete with activation regions (lightning bolts) used for this work. B) The surface-induced dissociation device used to dissociate the complexes. As commercialized by Waters Corp., the extractor and surface are connected by a jumper wire so that the surface and extractor are held at the same voltage. C) Schematic of the eMSion ExD device located in the pre-transfer ion guide. Panels A and B were adapted from reference 31 with permission from the Royal Society of Chemistry under a Creative Commons Attribution 3.0 Unported License.^31^ Panel C was adapted with permission from reference 32, copyright 2022 American Chemical Society.^32^

With each new instrument generation, end-users must optimize instrument tuning to meet their experimental needs. For the nMS community, this often means tuning instrument voltages to minimize ion activation in the source and other pre-selection quadrupole areas to maintain native-like biomolecule conformations; and in the post-activation areas to minimize the risk of secondary fragmentation.^48–51^ While the Waters cIM features a unique IM region that can be complex to tune, the Zenobi lab produced a helpful body of work related to that topic.^52^ In terms of activation regions, the Waters cIM instrument features multiple places for deliberate activation (Figure 1): the cone voltage between the StepWave and the source ion guide, the Trap cell,^53,54^ the IM pre-array,^54^ and the Transfer cell. To minimize activation prior to the selection quadrupole, the source region (ion source, StepWave, ion guide) should be carefully tuned to minimize unintentional activation and maintain native-like protein conformations. Between the selection quadrupole and the cIM, the Trap gradient, post-Trap bias, and helium cell should be tuned to minimize pre-IM activation, or to minimize secondary fragmentation after SID or Trap CID. To tune our instrument, we used Ubq’s measured CCS as a probe of pre-IM “hotness,”^55^ and SID of CRP to fine-tune for native protein complexes.^16^

**Figure 2.**
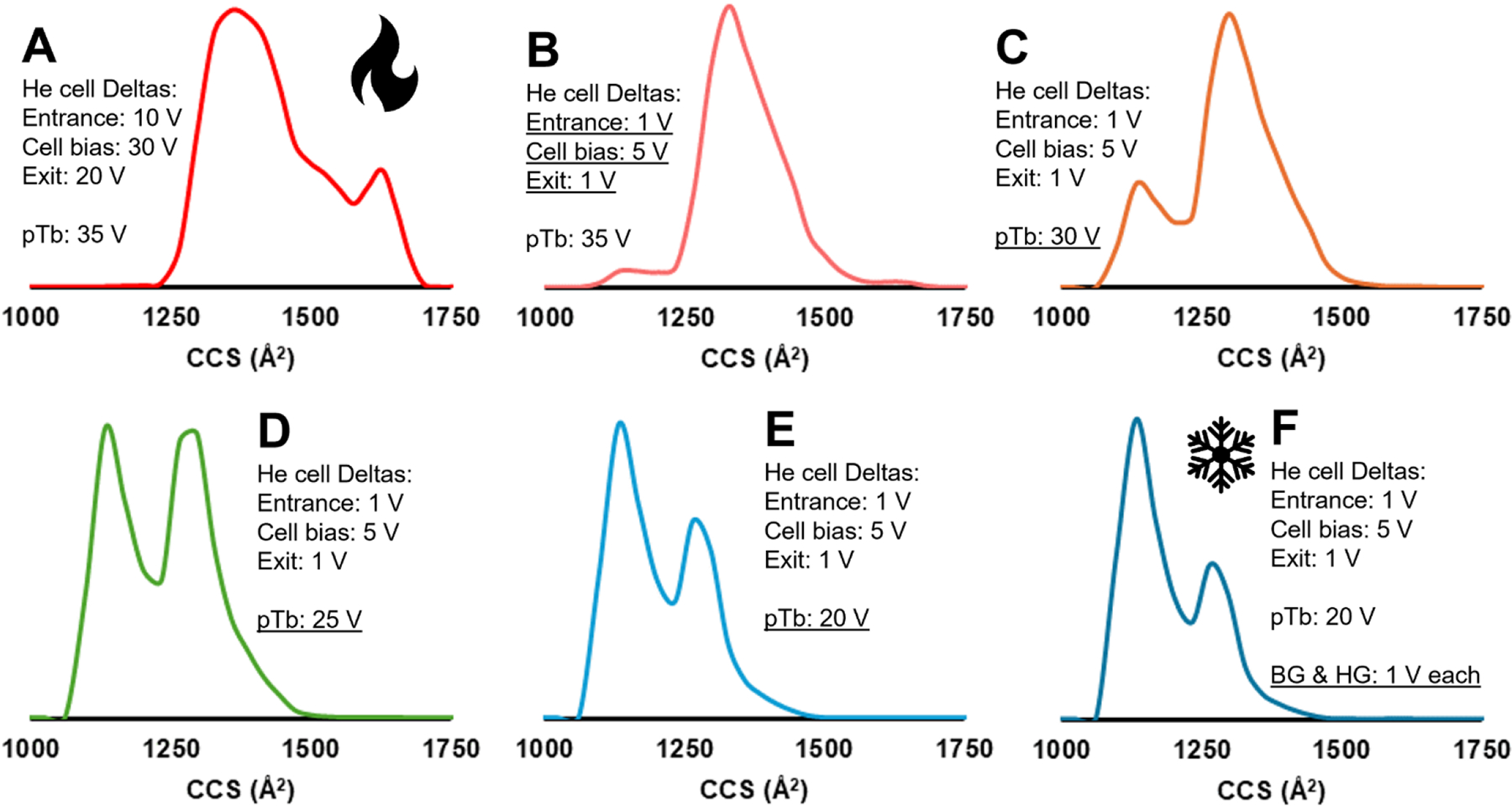
A – F) Collision cross-section distributions for Ubq 6+. He cell is the helium cell, pTb is the post-Trap bias, and BG and HG are the body gradient and head gradient, respectively, in the source StepWave. The underlined parameters highlight what is varied from one panel to the next. The flame icon indicates the “hottest” tuning profile, resulting in Ubq 6+ adopting extended conformations, while the snowflake indicates the “coldest” tuning profile, resulting in compact conformations. The red to orange to blue peak colors also indicate the transition from hot to cold tuning.

Depicted in Figure 2 are calibrated collision cross-section (CCS) distributions for Ubq 6+ (CCS calibration was achieved using Agilent ESI Tuning Mix as the calibrants,^56^ with IMSCal software).^57^ Panel A features the hottest (most activating) tuning, and panel F is the coldest (least activating) tuning, based on matches with CCS in the literature for Ubq.^55,58–63^ The relevant instrument parameters are listed in the figure. As the helium cell entrance, bias, and exit were tuned to have lower voltage deltas, the CCS distribution shifted from extended conformations between 1250 – 1750 Å^2^ to slightly more compact conformations between 1000 – 1500 Å^2^. As the post-Trap bias was lowered from 35 V to 20 V the CCS distribution shifted to favor the most compact conformational state, centered around 1100 Å^2^.^63^ When the StepWave body and head gradient deltas were lowered to 1 V apiece, a slight shift towards the most compact conformation is seen,^59,63^ though not nearly as dramatic of a shift as was seen in lowering the deltas and bias of the helium cell and post-Trap bias, respectively. It is possible that this slight shift could result from the larger, slower Ubq conformer being scattered in the He cell and not arriving at the detector. However, this could also affect the slower 5+ charge state as well, which is not observed when we compare relative intensities for 6+/5+ in the mass spectra under these different tuning conditions. In terms of absolute signal intensity, as the conditions are tuned from Figure 2A to 2F, the mass spectra indicate an approximate 50% signal intensity loss as the instrument conditions become cooler. Despite that loss the spectra are still easily interpretable with appropriate S/N. With minimized deltas in the StepWave and the helium cell, and minimized post-Trap bias, the least activating tune parameters were found for Ubq.

**Figure 3.**
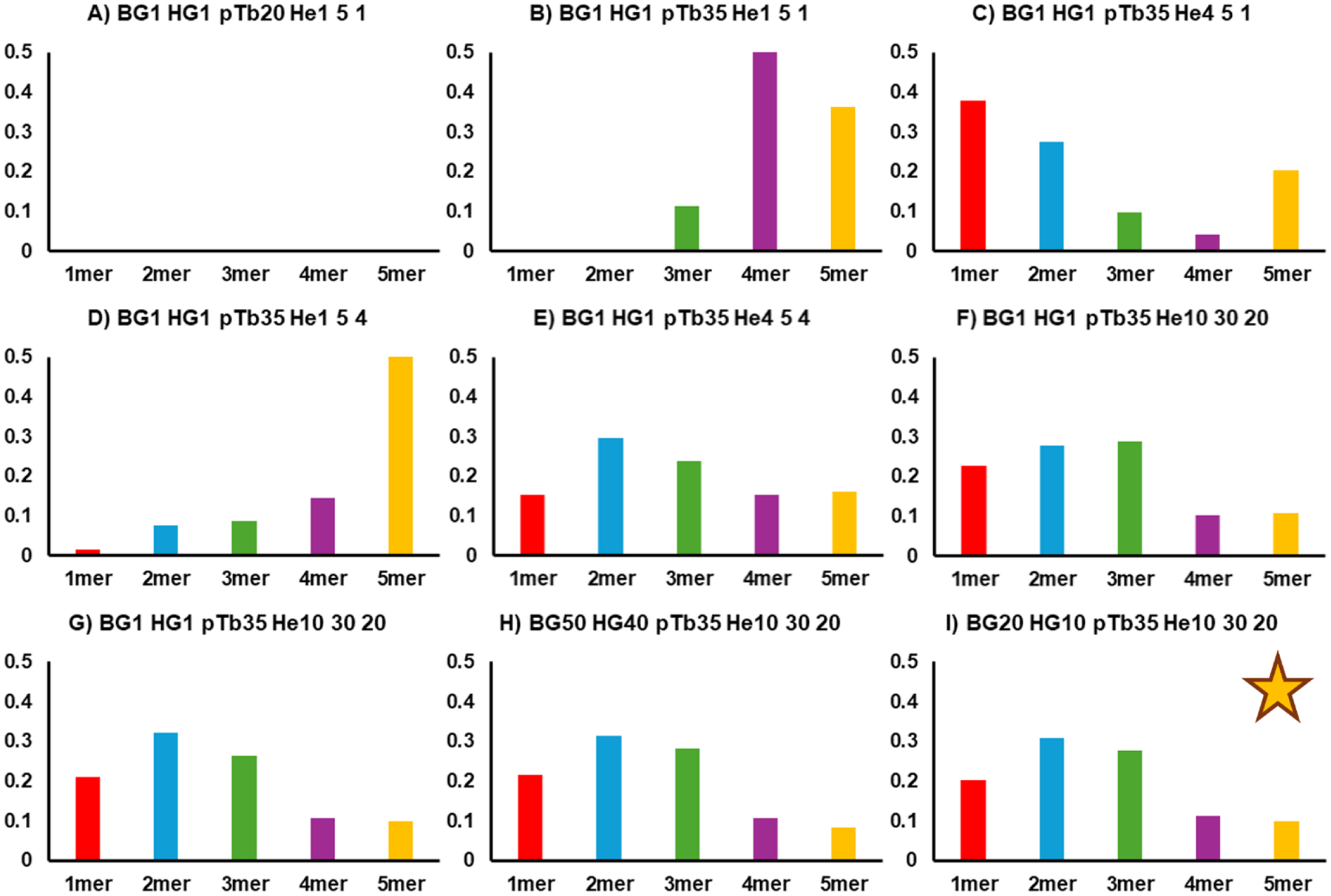
A – I) SID bar chart for 765 eV SID of CRP 17+ pentamer. The letter acronyms are the same as in Figure 2, with the input value listed directly after the acronym. In the case of the helium cell (He) the entrance, cell bias, and exit are listed from left to right. The yellow star on panel I indicates the tuning we used for our experiments, though similar results could be obtained with conditions listed in panels F-H as well. Panel E also appears to give optimal SID results, but the high m/z portion was absent from spectra and the mobiligrams, which is not desirable. Panels F and G have the same tuning parameters, but the pushes-per-bin was increased from 3 for panels A – F, to 4 for panels G – I. The terms 1mer, 2mer, etc. were used in place of monomer, dimer, etc. due to space constraints within the figure panels.

With these minimally activating tune settings, CRP was used to ensure good ion transmission after SID. Figure 3 depicts a series of SID bar charts for charge-reduced CRP (17+), in line with the charge state used for the data described later in the paper. With the least activating tune parameters for Ubq (Figure 2F), no ions were observed in the mass spectrum or mobiligrams (Figure 3A). Increasing the post-Trap bias from 20 to 35 V (panel B) resulted in evidence for trimer, tetramer, and pentamer, which suggests poor collection of complementary ions (monomer to accompany tetramer, dimer to accompany trimer). Additionally, the spectra and mobiligrams featured low ion intensities. Panels C – F were obtained by adjusting the indicated helium cell deltas and bias. Comparing these panels reveals that tuning the helium cell improperly can result in ion transmission bias, depending on the relationship between the helium cell entrance and exit deltas. Panel E appears to feature native-like SID for CRP, however, the high m/z region was missing from the mobiligram, which was concerning because complementary oligomers and charge states were not present when they reasonably should have been. These product ions appeared when the helium cell deltas were increased to the values in Panel F.

Panels G – I featured a pushes-per-bin value of 4, while panels A – F used a value of 3. The yellow star on panel I indicates the final tune settings we used for the SA, CRP, and TRAP experiments discussed below, except a pusher-per-bin value 0f 3 was used. Similar results could be obtained with the tune settings present in F – H. While it is important to consider different instrument configurations and detection strategies when comparing data acquired on different instruments, the Wysocki group has routinely shown that SID results are instrument agnostic, and that standard proteins should be used for benchmarking new SID installations.^15,16^ It is apparent when comparing the conditions in Figures 2 and 3 that what is optimal for ubiquitin (Figure 2F) is not ideal for CRP (Figure 3I). Because CRP is larger and features more non-covalent interactions than Ubq, the conditions that elongate Ubq do not have the same effect on CRP. This contrast highlights the importance of tuning an instrument for the analytes of interest and that no single tuning condition will preserve native-like structures for all proteins.

To confirm the suitability of our final tuning, an SID energy-resolved mass spectrum (ERMS) plot was obtained for CRP 18+, the charge state used by Harvey et al.^16^ The ERMS plot in Figure S1 features the dimer/trimer pair as the initial dominant SID product pathway, followed by monomer/tetramer. These pairs rise together in normalized intensity until dimer/trimer deviate due to secondary fragmentation of trimers to form monomer/dimer pairs and potentially dimer to monomer and tetramer to dimer-dimer or monomer-trimer, which also causes the monomer/tetramer pair to no longer track together. As the SID energy increases, monomer formation becomes the dominant SID pathway. These results clearly depict native-like SID fragmentation for the CRP pentamer, confirming that our tuning parameters (Figure 3I) are suitable for native-like analyses of protein complexes.

As discussed above, and as will be illustrated below, SID is an excellent tool for probing the “nativeness” of protein complexes. It can clearly indicate when instrument tuning is too harsh. Thus, SID of a standard protein complexes was key to confirming that our instrument tuning was minimally activating prior to SID and cIM.

### Experiment Types: isCA-IM and isCA-SID-IM

**Figure 4.**
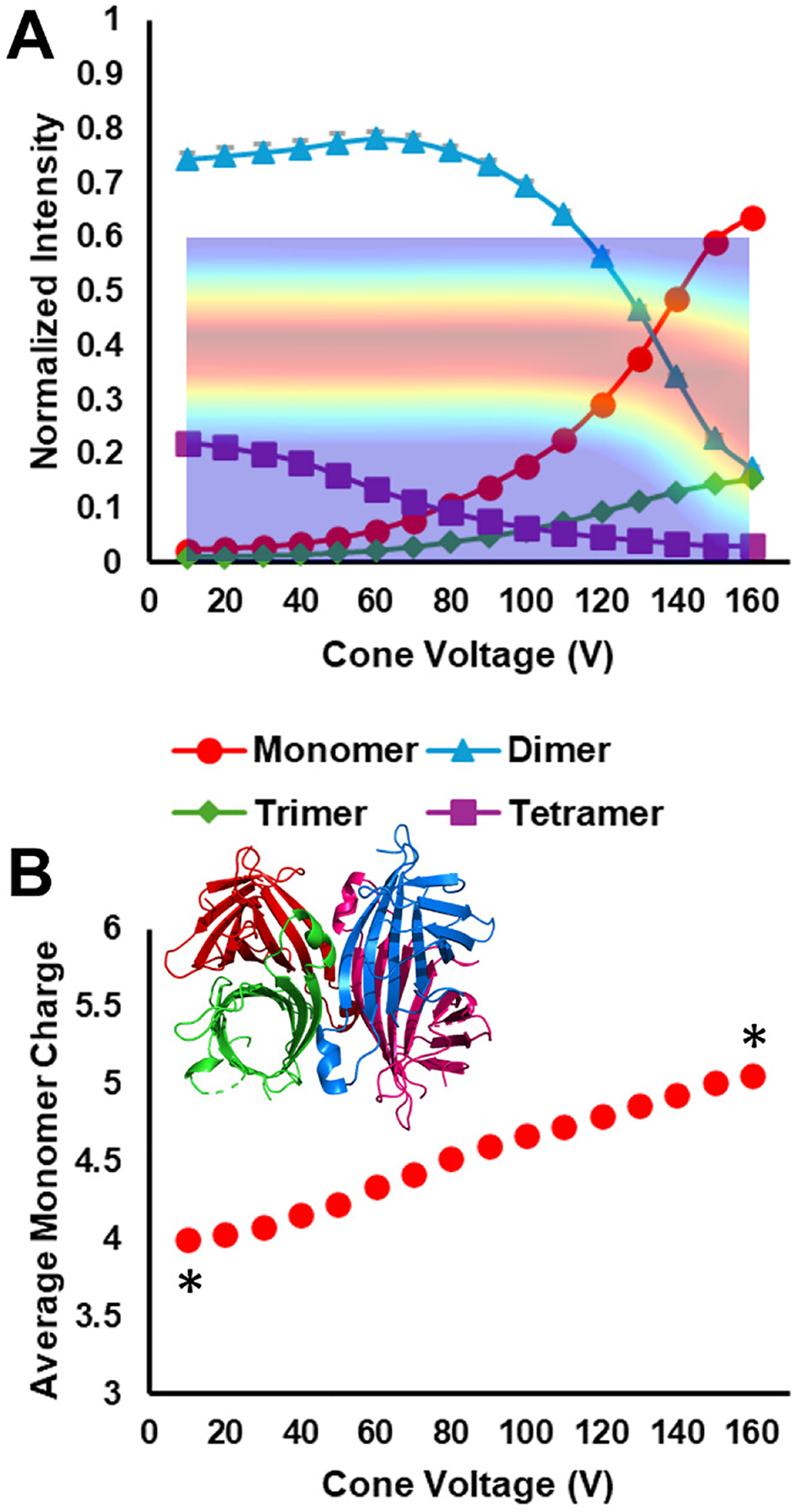
A) 11+ SA isCA_Δ_–SID_C (495 eV)_ plot with overlayed CIx plot. The implied y-axis of the overlay is drift time. Comparing the SID product trends and the CIx plot reveals product intensity shifts at lower voltages than resulted in drift time shifts within the CIx plot. B) SID monomer product average charge state. The asterisks indicate a statistically significant difference (*, p < 0.05) between monomer charge states at the indicated cone settings. The inset structure depicts the streptavidin tetramer (PDB 1SWB).

**Figure 5.**
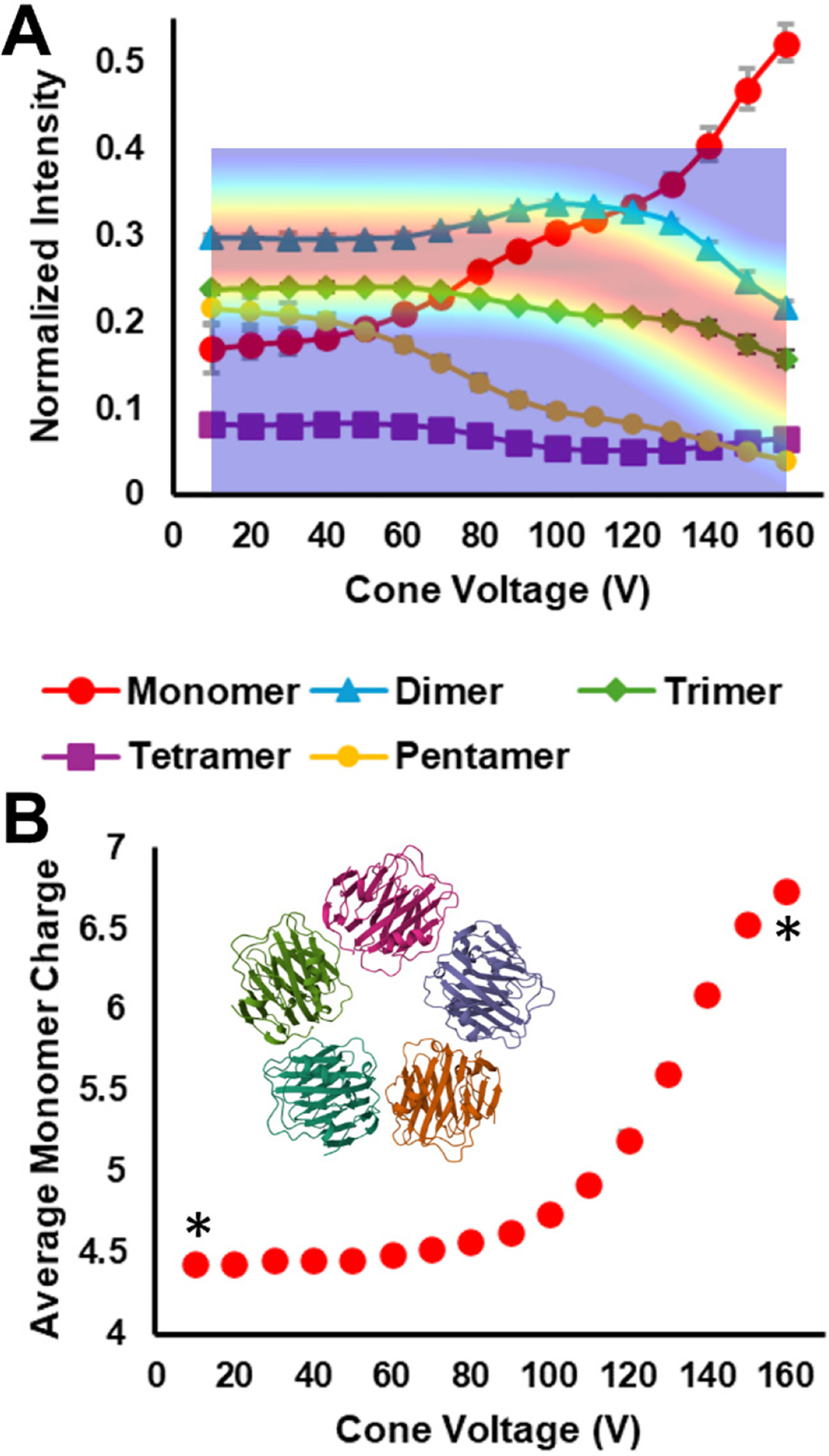
A) 17+ CRP isCA_Δ_–SID_C (765 eV)_ plot with overlayed CIx plot. The implied y-axis of the overlay is drift time. Comparing the SID product trends and the CIx plot reveals product intensity shifts at lower voltages than resulted in drift time shifts within the CIx plot. B) SID monomer product average charge state. Increased isCA shifts the SID pathway to favor monomer formation over the native-like dimer/trimer trend. Additionally, as the cone voltage increases, the monomer’s post-SID weighted average charge increases in a statistically significant way (*, p < 0.05). The inset structure depicts the CRP pentamer (PDB 1GNH).

**Figure 6.**
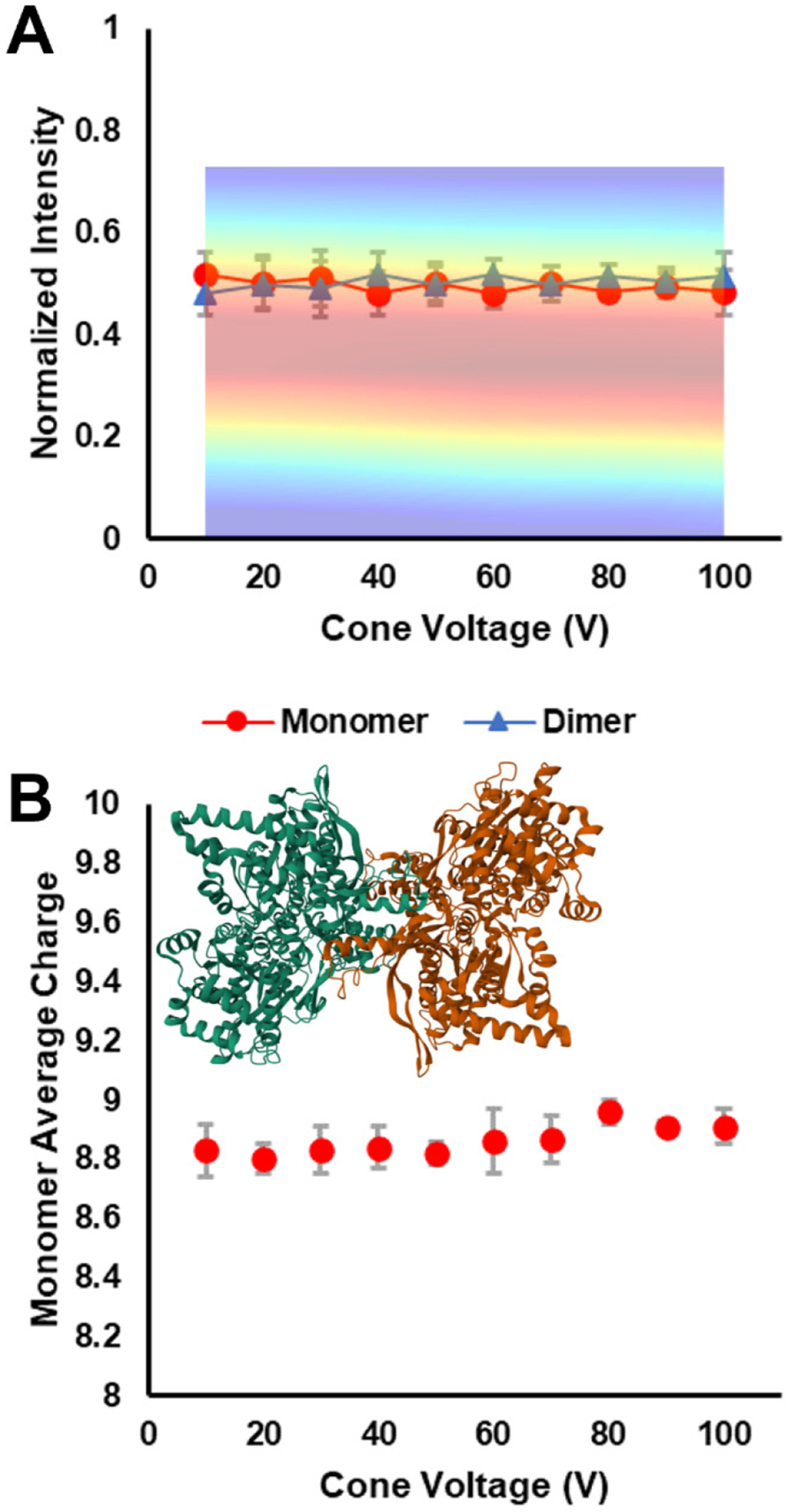
A) 21+ PhosB isCA_Δ_–SID_C (3990 eV)_ ERMS plot with overlayed CIx plot. The implied y-axis of the overlay is drift time. B) Monomer average charge state. Increased levels of isCA do not have a statistically significant effect on SID pathways nor on the monomer’s subsequent charge state. The inset depicts the PhosB dimer (PDB 1GPB).

**Figure 7.**
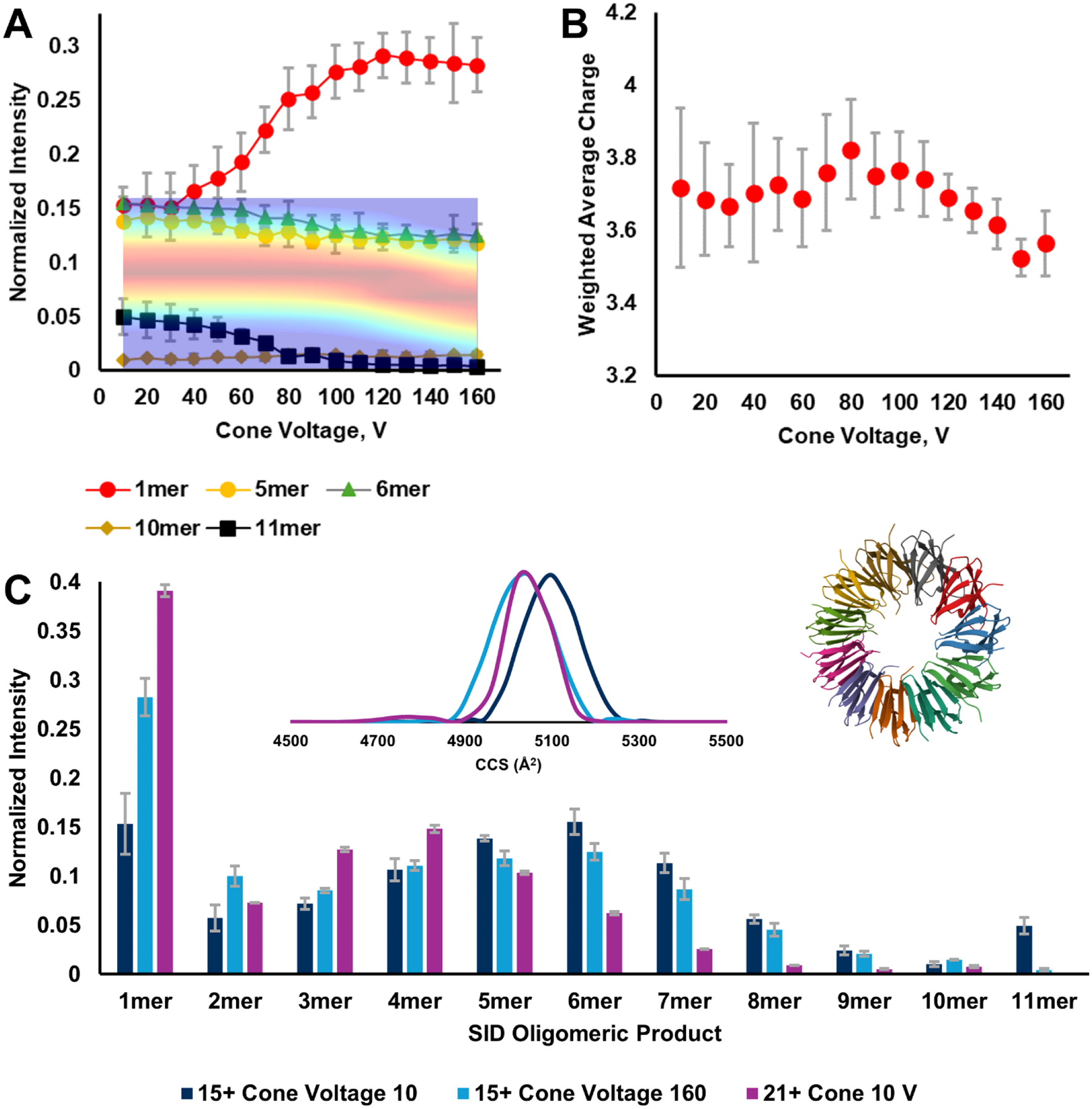
A) 15+ TRAP isCA_Δ_–SID_C (1050 eV)_ plot for a few indicated SID products, the remainder of which are found in Figure S9; the overlay is the TRAP 15+ CIx plot; B) SID monomer product average charge state, which shows no statistically significant trend. C) SID product comparison at 1050 eV of SID energy for the 15+ at two distinct cone voltages, and the 21+ at a low cone voltage. The left inset is the calibrated CCS for the indicated precursor charge states and their cone voltage settings. The CCS for the 15+ at high cone voltage and the 21+ are highly similar, suggesting they both feature a compact structure. The right inset structure depicts the TRAP bst 11mer (PDB 1GTN), with the tryptophan ligands removed.

Cone isCA collision-induced unfolding/collapse/restructuring (CIx) plots for all four charge-reduced protein complexes are found as overlays within panel A of Figures 4 – 7. The respective RMSD plots for the averaged triplicates are found in the SI (Figures S2 – S5). The RMSD values are high, ranging from 8 – 11. This is likely due to collecting the CIU data on separate days, which allows for pressure fluctuations that affect drift time. While CCS calibration could correct for such fluctuations, calibration would not affect the overall trends that are described hereafter. The plots obtained for SA (Figure 4A), and CRP (Figure 5A) reflect decreases in drift time as a function of increasing cone voltage. This suggests that the proteins’ quaternary structures were rearranged to smaller volumes due to isCA, as previously reported.^6,7,35,42,64–66^ CRP shows the most significant decrease in drift time at the highest cone voltages. Its structural rearrangement is partially attributable to its ring-shaped quaternary structure (inset, Figure 5) collapsing inward on itself, and possibly includes interactions between the elongating monomer chain with remaining complex (inferred from fragmentation patterns and charge states). Unlike monomeric proteins, protein complexes, especially when charge-reduced, are less likely to undergo major unfolding/expansion.^11,42,67,68^ The CIx plot for PhosB (Figure 6A and S4) does not possess noteworthy features that suggest significant quaternary rearrangement. Please note, the CIx plot overlay in Figure 6A covers isCA voltages from 0 – 100 V to align with the x-axis of the isCA – SID experiment. The full CIx plot, up to 160 V, for PhosB is found in Figure S4. While PhosB’s plot does not contain noteworthy features, the drift time does decrease linearly with increasing cone voltage. This appears to result from increasing amounts of collisional de-adducting as a function of isCA. Examining the mass spectra collected with cone settings of 20 V and 160 V (not shown) revealed a −14 *m/z* shift between the two cone settings, which corresponds to an ~300 Da mass loss for the 21+ charge state. Overall, while the isCA CIx plots (panel A in Figures 4 – 7) can be helpful in diagnosing source-induced quaternary structure rearrangements, this paper focuses on SID’s utility as a diagnostic tool, as described below in a series of experiments where the isCA was varied, as denoted by **isCA**_**Δ**,_ and the SID energy was held constant, as denoted by **SID**_**C**_. Based on that focus, the isCA_Δ_–SID_C_ plots in panel A of Figures 4 – 7 contrast with the overlaid isCA CIx plots to show how SID_C_ results show diagnostic differences that may not be reflected in a CIx plot. Note that the CIx plot involves activation by CID only (no SID).

#### Streptavidin isCA_Δ_–SID_C_.

Streptavidin is a tetramer that features a dimer-of-dimers topology (inset structure in Figure 4B).^16,69^ Thus, dissociation to dimers should be the preferred fragmentation pathway for native-like SID (Figure S6). This is the case for SA, as seen in Figure 4A, which reflects dimer as the primary dissociation pathway at low cone voltages. This trend is stable up to a cone voltage of approximately 90 V, at which point the normalized dimer intensity decreases as the cone voltage continues to increase. This suggests that either secondary fragmentation begins to occur when the sum of the isCA and SID activation exceeds a threshold, or the initial quaternary structure rearranges to a non-native-like structure that becomes apparent by the change in SID fragmentation and/or the change in drift time and product charge state. The CIx plot overlay in Figure 4A and S2, suggest that the drift time is stable until a cone voltage of 120 – 130 V. Between that point and the max cone voltage of 160 V, the drift time shifts to lower values, reflective of a more compact, non-native-like structure. Clearly, SID, as a diagnostic tool, is sensitive enough to detect excess energy deposition in the source, or rearranged quaternary structure, before ion mobility hints at the possibility that something has changed.^22^

As seen in Figure 4A, as the dimer’s contribution to normalized intensity decreases, the contribution from monomer and trimer increase. The slope of the monomer increase tracks with dimer decrease and is steeper than trimer increase, suggesting secondary fragmentation. The increase in trimer (and the portion of monomer produced from tetramer, not dimer) is a strong indicator that the weak subunit interfaces between dimer subunits, which are the most likely to be cleaved by SID, have been altered in a way that results in non-native SID, the mobiligram for which can be seen in Figure S7.

In comparison with SA’s CIx plot (Figure 4A and S2), SID was a more sensitive diagnostic for isCA than IM. The SID results showed significant shifts, due to post-SID secondary fragmentation, at lower cone voltages than were reflected in the CIx plot. Thus, reliance on IM to determine how harsh the ion source is, and how well the experiment conserved a protein complex’s native-like structure, could possibly lead to erroneous conclusions about the true nature of a complex with unknown topology.

Additional evidence for quaternary structure rearrangement by isCA is found in Figure 4B, which shows the monomer product’s average charge as a function of cone voltage. The statistically significant (*, p < 0.05) increase in average charge state suggests structural rearrangement that allows the monomer to abscond with more charge per mass than is typical under SID conditions.^69^ As demonstrated recently,^22^ this can happen if the monomer becomes elongated, as typical for CID. Despite the CIx plot showing a decrease in drift time of the tetramer, indicating quaternary structure collapse, the monomer’s increasing average charge state suggests that rearrangement involves monomeric expansion. This process is then measured and/or enhanced during SID, allowing for higher charged monomer to result.

#### C-reactive Protein isCA_Δ_–SID_C_.

We also examined the 17+ charge state of CRP under the same cone conditions. In contrast to SA, CRP is a cyclic pentamer with a ring-like topology (structure inset in Figure 5). The cavity in the middle provides volume for structural rearrangement not available to SA.^42^ IsCA clearly induces structural rearrangement (collapse), as seen in the overlay of Figure 5A and S3, where the drift time starts to decrease at about 100 V. This is 20 – 30 V lower than when SA’s drift time starts to decrease. Ostensibly, this suggests that SA may possess a more robust quaternary structure than CRP. However, CRP’s charge state is higher than SA’s, which would result in more energetic in-source ion-neutral collisions during isCA. CRP is also physically larger than SA,^70^ causing the pentameric protein to undergo more collisions than SA at the same source pressures. Regardless, isCA clearly induced structural rearrangement in CRP, as we’ve reported previously, and affected SID trends, as seen in Figure 5A.

CRP has a pentamer-of-monomers topology with equal interfacial areas between all subunits. This suggests that possible SID products include complementary pairs of monomer/tetramer and dimer/trimer, depending on the collision energy.^16^ At an SID energy of 765 eV, at low levels of isCA, dimer/trimer formation was the primary dissociation pathway, with monomer/tetramer also present, in line with previous reports from the Wysocki lab.^6,7,16^ This strongly suggests that the cyclic quaternary structure is intact and native-like.

As isCA becomes more energetic, the observed dissociation pathways change to favor monomer formation. An interesting region of the plot is between 80 – 110 V. In this region the monomer and dimer signals increase while the trimer and tetramer signals gradually decrease, and the residual pentamer signal dramatically decreases. These trends suggest post-SID secondary fragmentation, likely due to excess energy from isCA. As the ions undergo isCA, an amount of translational energy is converted to internal energy, leaving the ions internally hot. When these hot ions undergo SID, the sudden energy deposition results in native-like SID (dimer/trimer and monomer/tetramer formation), but the extra energy from isCA then drives the oligomeric product ions to dissociate further: trimer to monomer/dimer and tetramer to dimer/dimer and monomer/trimer. Since these secondary dissociation events have several pathways to monomer and dimer we see the commensurate rise in those respective signals. The trimer signal decreases overall since a portion of the initial trimer population dissociates to monomer/dimer while a new trimer population emerges since the initial tetramer product can form trimer as part of its secondary dissociation. This region shows how high amounts of isCA can have a dramatic effect on downstream tandem MS analyses, which would not have been apparent without carefully scanning isCA at a **fixed** SID potential. The end of this region coincides with the initial drift time shift in the overlayed CIx plot in Figure 5A, which suggests that quaternary structure rearrangement has been minimal and that increased internal energy from isCA is driving the shift in SID trends. At isCA voltages above 110 V, the monomer product becomes dominant, which aligns with the dramatic drift time shift in the CIx plot which indicates a collapsing quaternary structure.

Figure 5B tracks CRP’s SID monomer product average charge state as a function of cone voltage. Evident from the plot is the statistically significant (*, p < 0.05) increase in monomer average charge state with increased isCA, which agrees with similar work from the Wysocki lab in 2015, performed on a Waters Synapt G2.^6^ The interpretation given at that time is that the cone isCA process, at 160 V, both rearranges the protein such that the measured CCS is smaller (shorter drift time in our data), while also causing a monomeric subunit to partially elongate but remain non-covalently bound to the remaining subunits. The SID process then allows that elongated monomer to abstract additional charge as it leaves the protein complex. This is also consistent with what we observed for SA. Returning to the interesting regions between 80 – 110 V, the average monomer charge state increases at a faster rate than it did between 10 – 80 V. Based on the discussion above, this could be due to the increase in secondary fragmentation from energy deposition during isCA followed by SID. As the CIx plot reflects quaternary structure collapse, the average monomer charge state appears to approach a rollover point to a constant value. Unfortunately, this rollover point is not completed due to the upper limit on the cone voltage.

#### Phosphorylase B isCA_Δ_–SID_C_.

We also included PhosB, a homodimer whose CIx data are presented in Figure 6A and S4, and SID data in Figure 6. In terms of CIx, the plot only shows a slight decrease in the drift time as a function of cone voltage, otherwise there are no distinct features that suggest quaternary structure rearrangement. This drift time decrease does suggest mass loss, which is confirmed when the deconvolved and centroided mass spectra collected with cone settings of 10 V and 160 V are compared for the 21+ charge state. The spectrum collected at a cone = 100 V features a deconvolved mass that is ~156 Da lower than that collected at cone = 10 V. At the highest cone voltage, 160 V, the deconvolved mass is ~300 Da lower than that collected at cone = 10 V. While structural rearrangement is not evident, the higher cone voltage clearly helped with in-source protein cleanup, i.e., loss of adducts such as (H_2_O), (NH4+) and (CH3COO-).

In terms of SID, the instrument has a total voltage limit of 300 V across all activating regions, including the cone voltage, SID/Trap, and Transfer regions. Thus, with an SID voltage of 190 V, the cone voltage could only be tuned up to ~100 V. Hence, the x-axis of Figure 6 does not reach the same cone voltage as used in Figure S4. For SID of PhosB, monomer formation will be the favored dissociation pathway, if the SID energy is sufficiently high. Previous work in our lab found that the ratio of product monomer and surviving precursor dimer is about 50:50, even at the highest SID energies.^16^ Taking that into account, and factoring in the CIx results, we anticipated that increased isCA would not affect the SID trend since the only SID product is the monomer subunit. As seen in Figure 6A, this is indeed the case. Regardless of the cone voltage, no significant changes occur in the SID trend. In striking comparison to the results obtained for SA, and CRP, this SID trend suggests no measurable structural rearrangement took place.

Additional evidence that suggests minimal to no quaternary structure rearrangement is found in the monomer product’s average charge. As depicted in Figure 6B, the average charge remains steady as a function of cone voltage. This was somewhat surprising, since isCA could have caused the monomeric subunits to elongate in a way that more charge could be placed on the monomers during SID, even if the SID trend was not disrupted. This apparently did not happen. To confirm this, the mobiligrams at each cone voltage were compared to each other and no difference in mobility arrival time was observed as a function of cone voltage. Given the fact that isCA was limited to low voltages when coupled to SID, it is unsurprising that no quaternary structure rearrangement was observed for PhosB.

#### TRAP isCA_Δ_–SID_C_.

In 2005 Ruotolo, Robinson, and coworkers published a convincing report that native-like protein quaternary structure could be maintained in the gas phase after nESI from ammonium acetate.^71^ The report was founded on ion mobility measurements for TRAP from *Bacillus subtilus*. Careful CCS calibration and comparison with predicted CCS values for model structures indicated that the 21+ charge state likely featured a collapsed, globular, 11mer structure while the 19+ charge state was likely an intact cyclic ring. Since then, the Wysocki lab has published SID data comparing the 21+, 15+, and 16− charge states.^7^ The 21+ and 15+ had distinctly different SID fingerprints, with the latter showing native-like fragmentation for a ring-like quaternary structure. The 16− had similar features to the 15+. This distinct SID behavior persuaded us to include TRAP here as well, to illustrate the effect of overly energetic isCA on an undecameric protein complex.

The cone CIx plot for the 15+ charge state is found in Figure 7A and Figure S5. Based on the drift time, the quaternary structure appears to be stable at cone voltages below 120 V. However, the drift time decreases to a new stable feature at cone voltages above 120 V. This suggests that isCA causes the cyclic protein complex to collapse. The ultimate effect of isCA on SID is seen in Figure 7A, which shows a few selected SID products. The full plot with all SID products can be seen in Figure S8. At cone voltages below 60 V and with a **fixed** SID energy of 1050 eV, the most prevalent SID products are the monomer, pentamer, and hexamer subunits, followed by residual 11mer precursor. As seen in Figure 7C (blue bars), the other complementary oligomeric pairs (1/10, 2/9, 3/8, 4/7) seem to have complementary signal intensities, which should be due to native-like fragmentation. As isCA becomes more aggressive Figure 7A shows a significant shift in SID behavior at a cone voltage of 50 – 70 V, a trend that seems to level off by a cone voltage of 100 V, when the CIx plot barely starts to suggest the possibility of structural rearrangement. The monomer signal is this region, cone voltage 50 – 100 V, appears similar to the monomer trend for SA (Figure 4A) and CRP (Figure 5A), which suggests that secondary fragmentation due to energy deposition from isCA and SID is at play here as well. As seen in Figure 7C (red bars) at the highest cone voltage (most severe isCA), the SID product distribution shifts towards lower oligomeric states, more reflective of non-native fragmentation behavior as captured for the 21+ charge state at the same SID energy (Figure 7C gold bars).

As shown in Figure 7B, the SID monomer product’s average charge shows limited variation with increasing isCA, as opposed to the increasing charge states with increasing isCA observed for SA, AV, and CRP, and previously reported by our lab.^6^

Figure 7C compares SID product intensities of 15+ TRAP at two different cone voltages, and the 21+ charge state at low cone voltage. Based on the above mentioned papers,^7,71^ it was reasonable to expect that the 21+ charge state (purple bars) would feature non-native-like SID. This is clearly reflected in Figure 7C, where the SID oligomeric product distribution is shifted towards lower oligomeric states, whereas the 15+ charge state at low cone voltage (dark blue bars) appears to have more balance across the oligomeric states, reflecting native-like partitioning when two interfaces are cleaved in an 11mer protein complex. The 15+ charge state at the highest cone voltage (light blue bars) is shifted to favor lower oligomeric states, similar to the 21+ charge state. This clearly shows that a high amount of isCA caused 15+ TRAP to adopt a collapsed quaternary structure, resulting in non-native-like SID.

Lastly, the inset of Figure 7C is the CCS distribution for the same three precursor ion conditions (charge states 15+ at cone 10 and 160V, and charge state 21+ at cone 10V). Because we are comparing two different charge states, we felt it appropriate to calibrate TRAP’s CCS. We did so using IMSCal,^57^ and a CCS database composed of protein complexes under native and charge-reducing conditions.^70^ The calibrants selected included: alcohol dehydrogenase, c-reactive protein, and streptavidin, which were prepared as described for other proteins complexes above. These calibrants were sprayed from 200 mM ammonium acetate and 160/40 mM ammonium acetate/TEAA.

As seen in the inset of Figure 7C, there is a size difference between the 15+ and 21+ at a cone voltage of 10 V that suggests the 15+ is more expanded, perhaps cyclic, while the 21+ is indeed globular. When the 15+ complex undergoes isCA at a cone voltage of 160 V, the CCS decreases to a range that matches the CCS for the globular 21+. One possible reason for this match is that isCA caused the 15+ to collapse from a ring-like structure to a globular structure, similar to that possessed by the 21+. We note that in comparison to the CCS values published by the Robinson lab in 2005,^71^ our CCS values for TRAP are smaller by about 9%. This could be due to the advancement that traveling wave ion mobility has experienced since 2005, especially in terms of better understanding of its physical implementation, theoretical underpinnings,^72^ application,^73^ and improved experimental CCS calibration.^57^

### Experiment Type: isCA-IM-ECD

To explain why we observed these SID trends as a function of isCA, we find it helpful to think about the subunit interfaces within the protein complexes. Previous work from our lab showed that SID will cause native-like protein complexes to dissociate along their weakest subunit interfaces. By this we mean the interfaces with the lowest interface area, fewest salt bridges, fewest hydrogen bonds,^16^ and hydrophobic surface area.^19^ When the protein’s quaternary structure is disrupted by isCA, it is very likely that the subunit interfacial areas, salt bridges, and hydrogen bonds were disrupted along the way to low energy, non-native-like structures. These rearranged interfaces and interactions then influenced subsequent SID pathways.

To characterize how subunit interfaces were possibly rearranged by isCA, we employed top-down electron capture dissociation (TD ECD). ECD cleaves the peptide N-C_α_ bond, typically resulting in c/z product ion types.^74^ For native-like proteins, the released product ions can be indicative of protein secondary, tertiary, and even quaternary structure. Additionally, the obtained products can be sensitive to pre-ECD collisional activation, thus this technique can also diagnose rearranged protein structures^.26,30,33,75,76^

Figures S9 and S10 show SA’s ECD sequence coverage at cone voltages of 20 V and 160 V, respectively. We note that the depicted sequence includes an N-terminal methionine residue. The SA commercially available from Thermo Scientific does not have all N-terminal methionine residues cleaved, which is evident in the mass spectrum. Because the tetramer likely forms in a stochastic manner, there are mass spectral peaks that correspond to 0, 1, 2, 3, and 4 conserved methionine residues on the complex, with individual monomer subunits carrying 0 or 1 Met. Thus, we used both possible sequences, with and without the leading Met, but choose to display the ECD data featuring the leading Met. Cleavage sites were similar between the two protein sequences.

Using Proteins, Interfaces, Structures and Assemblies (PISA) (https://www.ebi.ac.uk/pdbe/pisa/),^77^ an interface analysis was performed on PDB 1SWB, the SA tetramer. Identified interfacial residues are located within SA’s anti-parallel β-sheet. In Figures S8 and S9 these residues are highlighted in tan. The residues between the highlighted regions belong to the β-sheet’s hairpin turns and were not included as interfacial residues by PISA. Returning to Figures S8 and S9, a cone voltage of 20 V resulted in 24% sequence coverage, not including internal fragments. The sequence coverage was 53% at a cone voltage of 160 V. Since the SID data indicate that isCA clearly rearranges the quaternary structure, possibly by causing monomer subunits to elongate, the uptick in sequence coverage was unsurprising. Next, we should note that the increased sequence coverage came from the N-terminus, and the interface between subunits, especially between residues 75 – 120. This strongly suggests that isCA disrupted protein tertiary structure by elongating the N-terminal domain and rearranged the quaternary structure along the strong monomer-monomer interfacial areas. This evidence validates our conclusion that the SID results in Figure 4 reflect interfacial changes between subunits, which was also suggested by the increase in average monomer charge state.

ECD analysis at cone voltages of 20 and 160 V was also attempted for 17+ CRP, 21+ PhosB, and 15+ TRAP, however, no fragments were obtained for these complexes. We did observe extensive gas phase electron capture charge reduction (ECCR)^78^ with the average charge state being 12+ for CRP, with a precursor charge state range of 8+ through 17+. For PhosB, the average charge was 14+ with a range encompassing 7+ through the 21+ precursor. The average charge for TRAP decreased to 12+, with a range of 6+ to 15+. We did attempt to tune the Transfer collision energy and Transfer cell pressure to release ECD fragments, but those efforts did not result in ECD fragments being released from the protein complexes. We also attempted to tune the transfer RF amplitude to help capture possible ECD fragments, but to no avail. As we checked PDB 1GNH for CRP we found that the N-terminus is solvent accessible in the native structure while the C-terminus is buried in an interfacial interaction. For PDB 1GPB for PhosB both N- and C-termini are solvent accessible, while 1GTN for TRAP features N- and C-termini that are involved in interfacial interactions, some of which are present as hydrogen bonds. Because charge reduction without observed fragmentation was observed for CRP, PhosB, and TRAP, they can be classified as ‘group II’ following the Loo lab’s designation scheme, which suggests that inter- and intra-subunit interactions limit the number of fragments that can be obtained after ECD.^26^ For CRP, whose N-terminus was solvent accessible, it is possible that ionization into the gas phase resulted in the N-terminus becoming protected by new non-covalent interactions during desolvation, thereby limiting c-type fragmentation. Additionally, we suppose that CRP’s higher mass, compared to SA, could have contributed to low kinetic energy that Transfer CID could not overcome to liberate fragments. Thus, a heavier collision gas, such as argon or xenon, could increase the collision energy enough to free the ECD fragments from the complex or AI-ETD could be attempted.^79^

## CONCLUSION

This paper defines conditions to be used to keep protein complexes “native-like” in a cyclic IMS (q-IM-TOF) modified to include SID and ExD capabilities. Using Ubq 6+, with a goal of achieving a compact conformation that is considered in the literature to be native-like, we tuned the pre-IM voltage gradients to be as non-activating as possible. During this process we identified the post-Trap ion guide bias and the helium cell as the most activating regions, while adjusting the StepWave voltages resulted in minimal CCS changes. While such tuning was ideal to maintain Ubq’s native-like structure, the shallow voltage gradients resulted in poor SID product transmission for the larger standard protein complex CRP. We then systematically increased the post-Trap ion guide bias and the helium cell entrance, exit, and bias voltages to obtain good SID product ion transmission for this larger, more structurally stable protein complex, noting that the new conditions did not cause non-native fragmentation behavior. Taking lessons from Ubq, we maintained the instrument’s source, including the StepWave and cone voltage at low values as an added measure to preserve native-like structure for a range of standard protein complexes. We then systematically demonstrated that using the cone voltage setting for isCA to remove non-covalent adducts from protein complexes can result in both internal heating resulting in secondary fragmentation and, at higher energies, quaternary structure rearrangement. These effects were illustrated by using SID product ion distributions, CIx mobiligrams, and ECD. Secondary fragmentation, resulting from the additive nature of isCA and SID, altered fragmentation patterns and highlighted the importance of minimizing ion activation prior to the desired tandem MS steps even at isCA energies where drift time shift is not yet apparent. The approach reported here, that uses SID, CIx, and/or ECD of standard protein complexes,^80^ will be used to characterize native-like behavior in other instrument platforms in the future, to establish a standard “native” tuning protocol for new instrument acquisitions, released instrument modifications, or new practitioners of native MS.

## Supplementary Material

SI Roughhousing Ions

Supporting Information

The Supporting Information is available free of charge on the ACS Publications website.

SID energy-resolved mass spectrometry plot for CRP 18+; averaged CIx plots for SA, CRP, PhosB, and TRAP and their respective RMSD plots, sample SID mobiligrams illustrating native and non-native SID results, ECD sequence maps for SA at different cone voltages (docx)
