# Supplementary material for: Roughhousing with Ions: Surface-Induced Dissociation and Electron Capture Dissociation as Diagnostics of Q-Cyclic IMS-TOF Instrument Tuning Gentleness
Source: J Am Soc Mass Spectrom. Author manuscript; Available in PMC 2026 Jan 1. (PMC12318544; doi:10.1021/jasms.4c00417)
Supplement: SI Roughhousing Ions [file NIHMS2093359-supplement-SI_Roughhousing_Ions.pdf]

Supporting Information

Roughhousing with Ions: Surface-Induced Dissociation and Electron Capture Dissociation as Diagnostics of Q-Cyclic IMS-TOF Instrument Tuning Gentleness

Andrew J. Arslanian,[1,2] and Vicki H. Wysocki*[1,2]

[1]Native MS Guided Structural Biology Center, The Ohio State University, Columbus, Ohio, 43210, United States

[2]Department of Chemistry and Biochemistry, The Ohio State University, Columbus, Ohio, 43210, United States

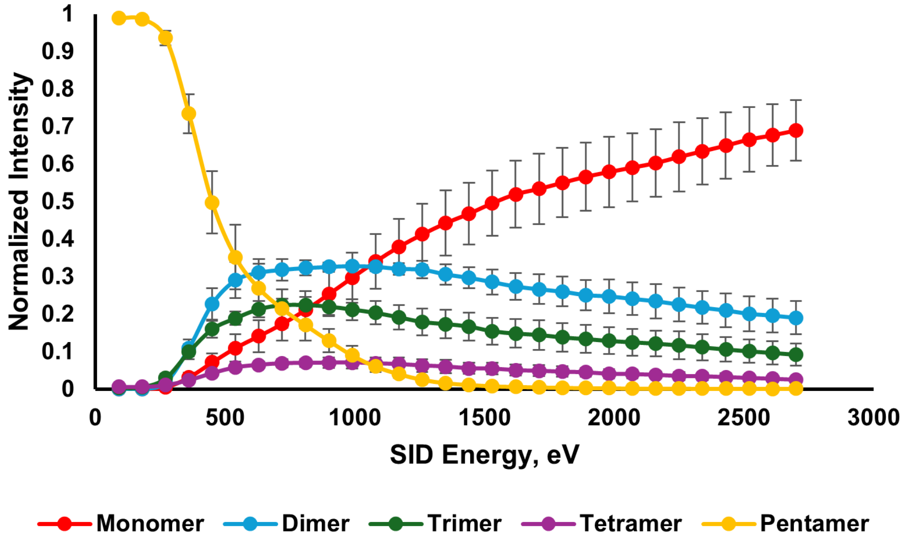

Figure S1: SID energy-resolved mass spectra (ERMS) plot for CRP 18+. This ERMS plot depicts native-like SID fragmentation for the CRP pentamer: the dimer/trimer pair rises together before splitting apart, likely due to secondary fragmentation driving the trimer to form monomer/dimer. The monomer/tetramer initially rise together, then monomer increases as SID deposits enough energy dissociate the trimer into monomer/dimer. As SID energy increases, monomer becomes the dominant product. This behavior is consistent with the ERMS plot published in the SI for reference 1.[1]

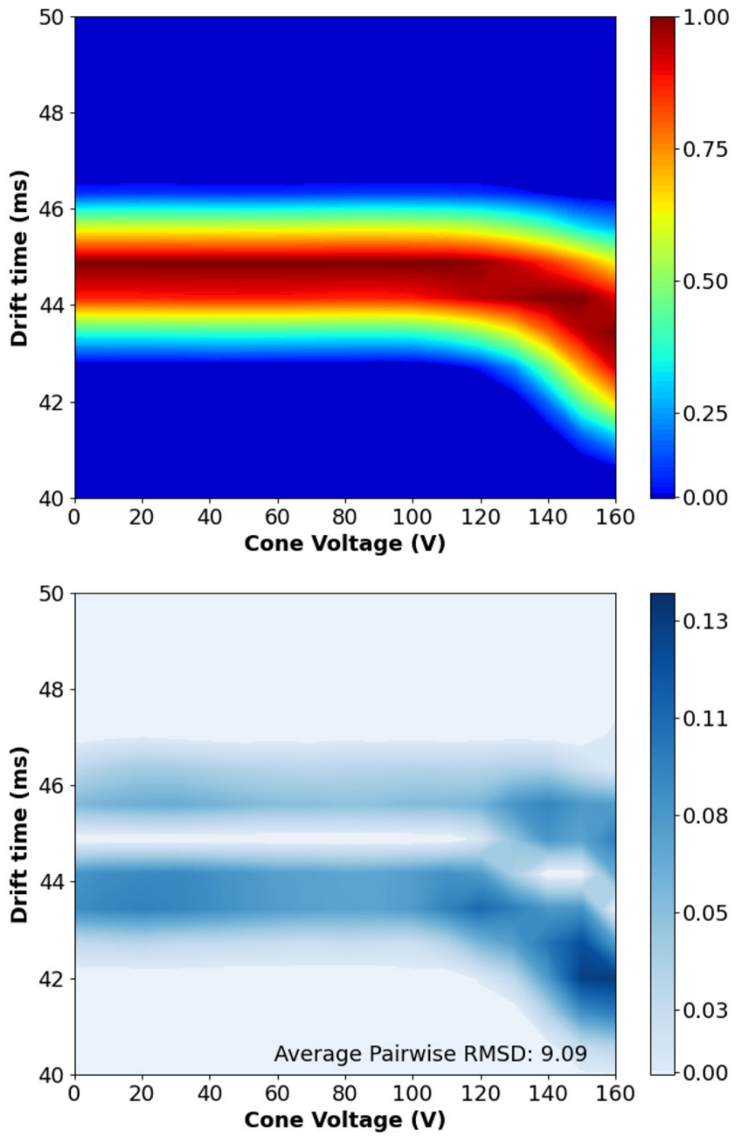

Figure S2. CIx plot and standard deviation for 11+ streptavidin. The drift time shifts to lower values at high cone voltage settings. This suggests that the tetrameric quaternary structure has collapsed.

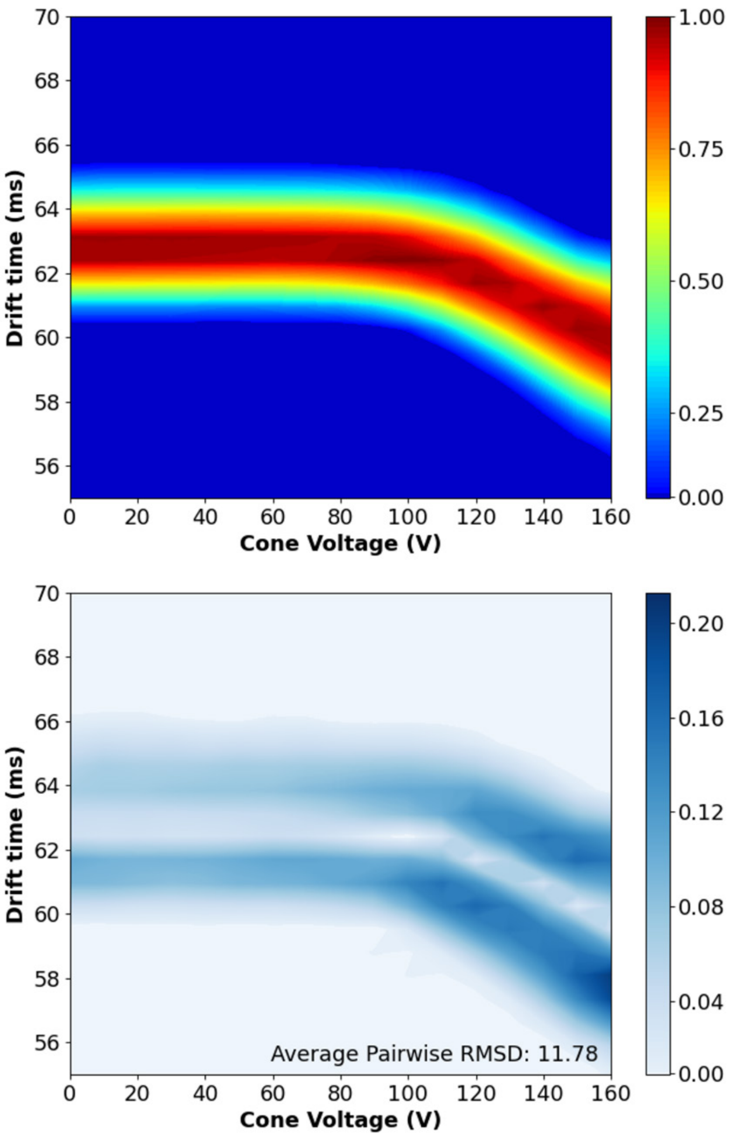

Figure S3. CIx plot and standard deviation for 17+ CRP. The significant drop in drift time is likely attributable to the ring-shaped quaternary structure collapsing upon itself.

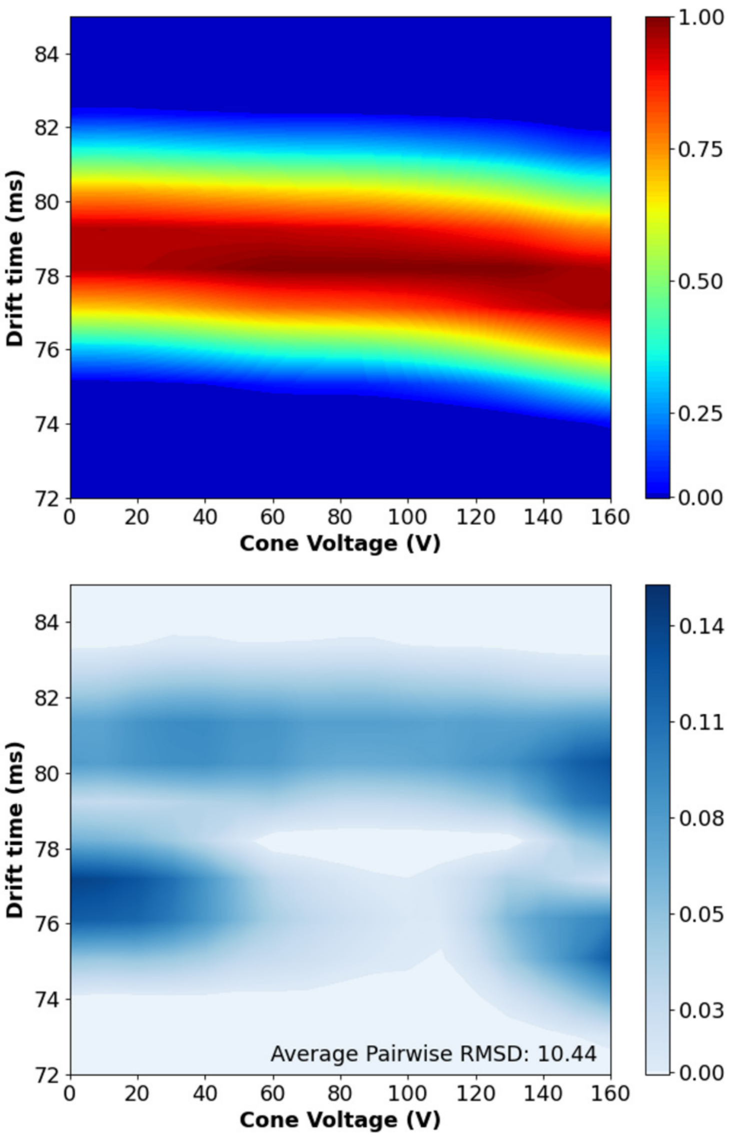

Figure S4. CIx plot and standard deviation for 21+ PhosB. While the drift time trends downward as the cone voltage increases, there are no noteworthy features that suggest source-induced quaternary structure rearrangement.

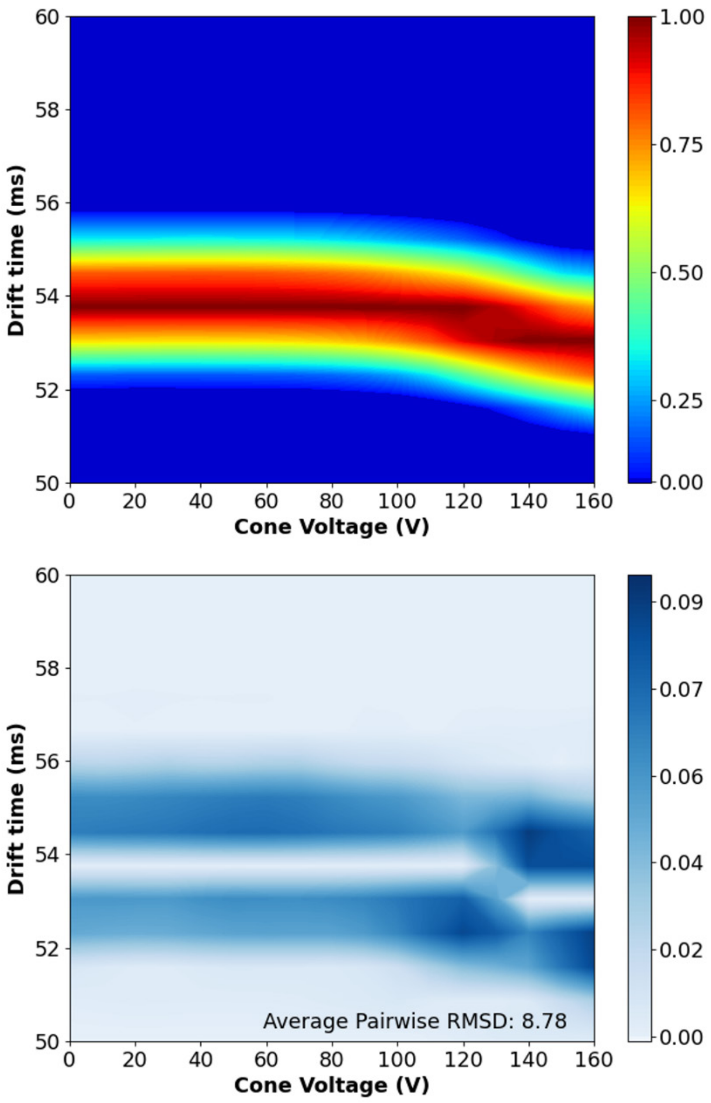

Figure S5. CIx plot and standard deviation for 15+ TRAP bst. The complex appears to suffer quaternary structure collapse at cone voltages higher than 120 V.

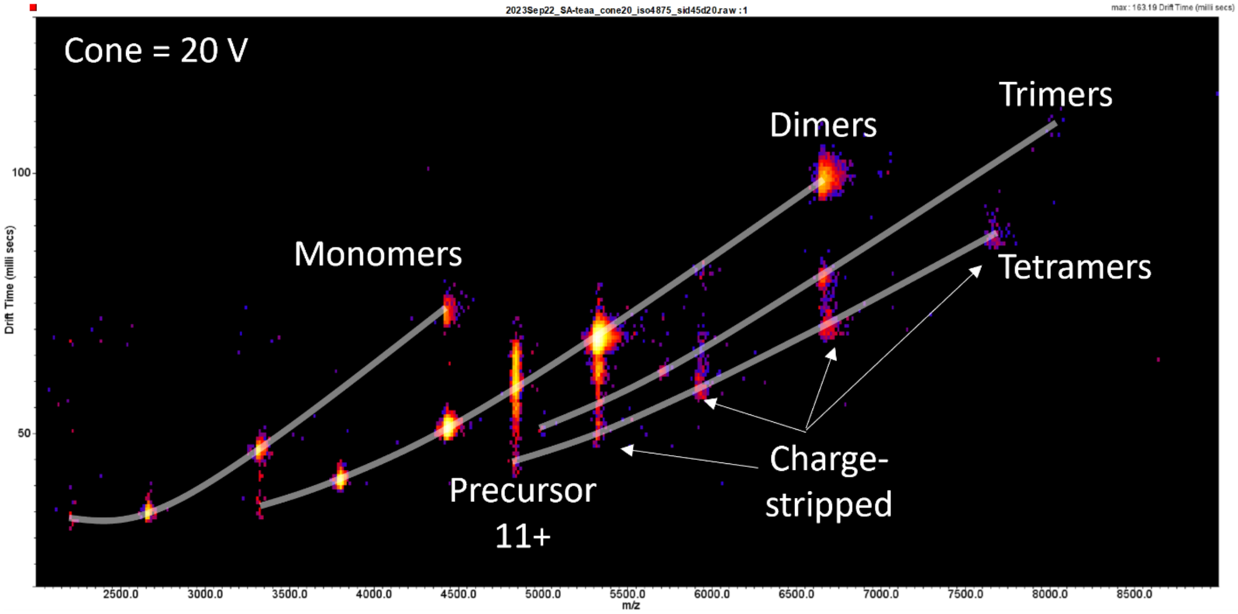

Figure S6: mobiligram for SA 11+ after SID at 495 eV, with a sampling cone voltage of 20 V. This fragmentation pattern is regarded as native-like since the primary SID pathway resulted in dimer formation.

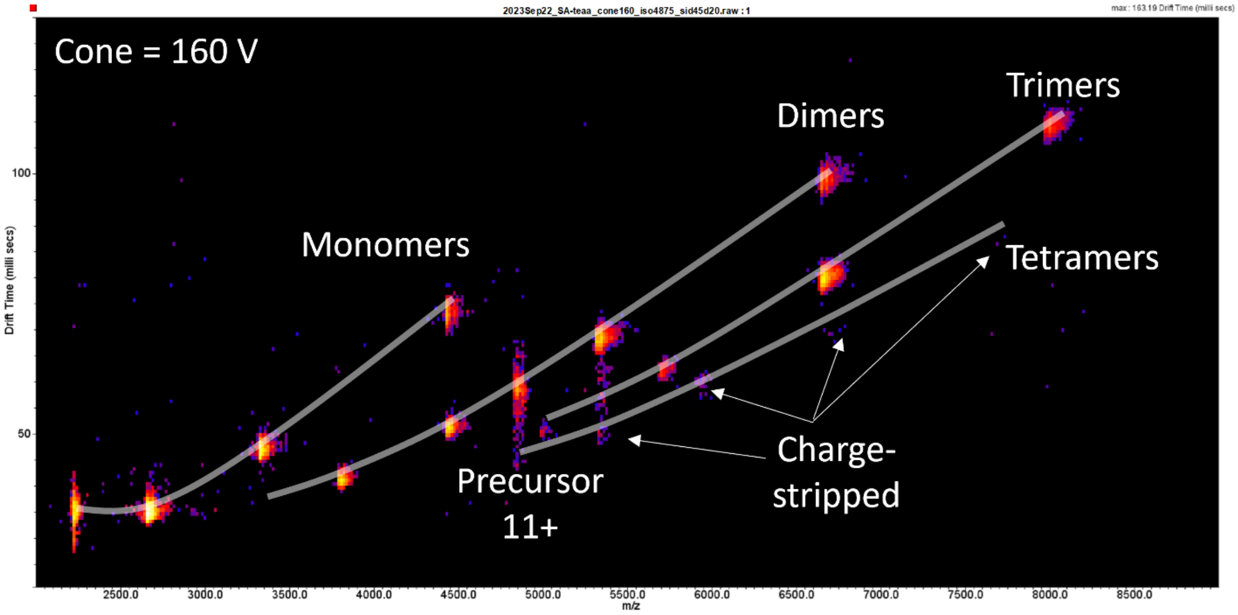

Figure S7: mobiligram for SA 11+ after SID at 495 eV, with a sampling cone voltage of 160 V. This fragmentation pattern is regarded as only partially native-like since the primary SID pathway resulted in reduced dimer and increased monomer/trimer formation. Within the monomer trend line it is obvious that the 5+ and 6+ charge states became more intense than was present in Figure S6 at a cone voltage of 20V.

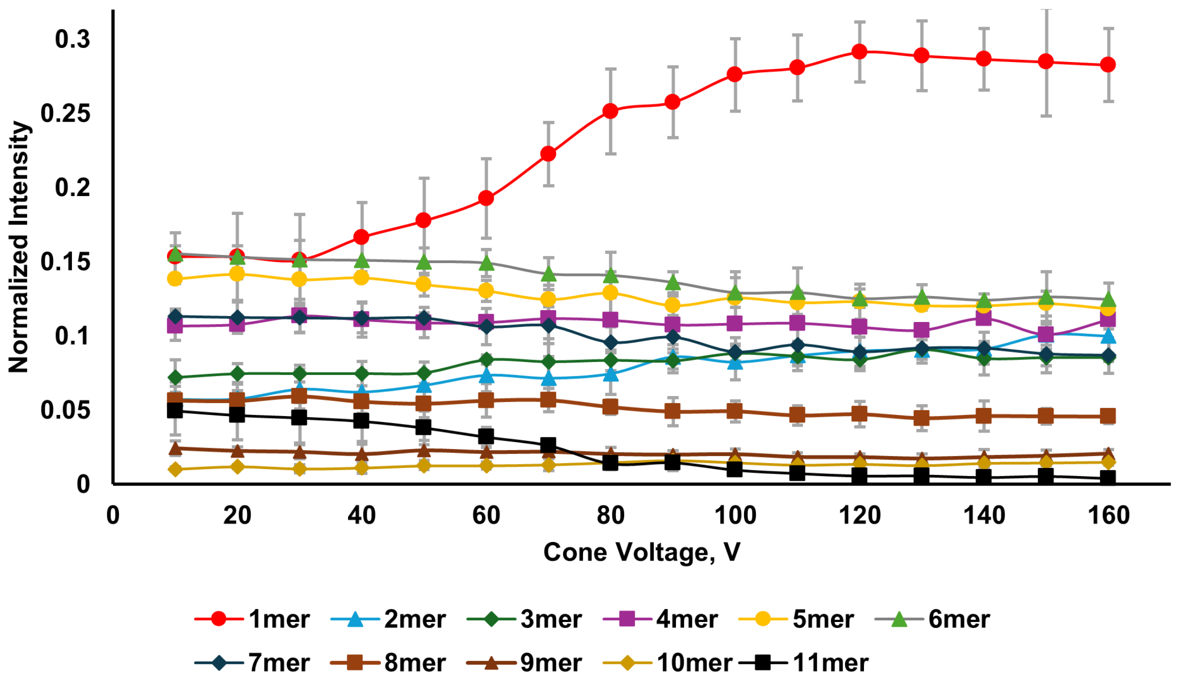

Figure S8. isCA – SID (1050 eV) plot of TRAP bst. Increased isCA shifts the preferred dissociation pathway towards monomer formation, likely due to isCA causing the 11mer ring structure to collapse.

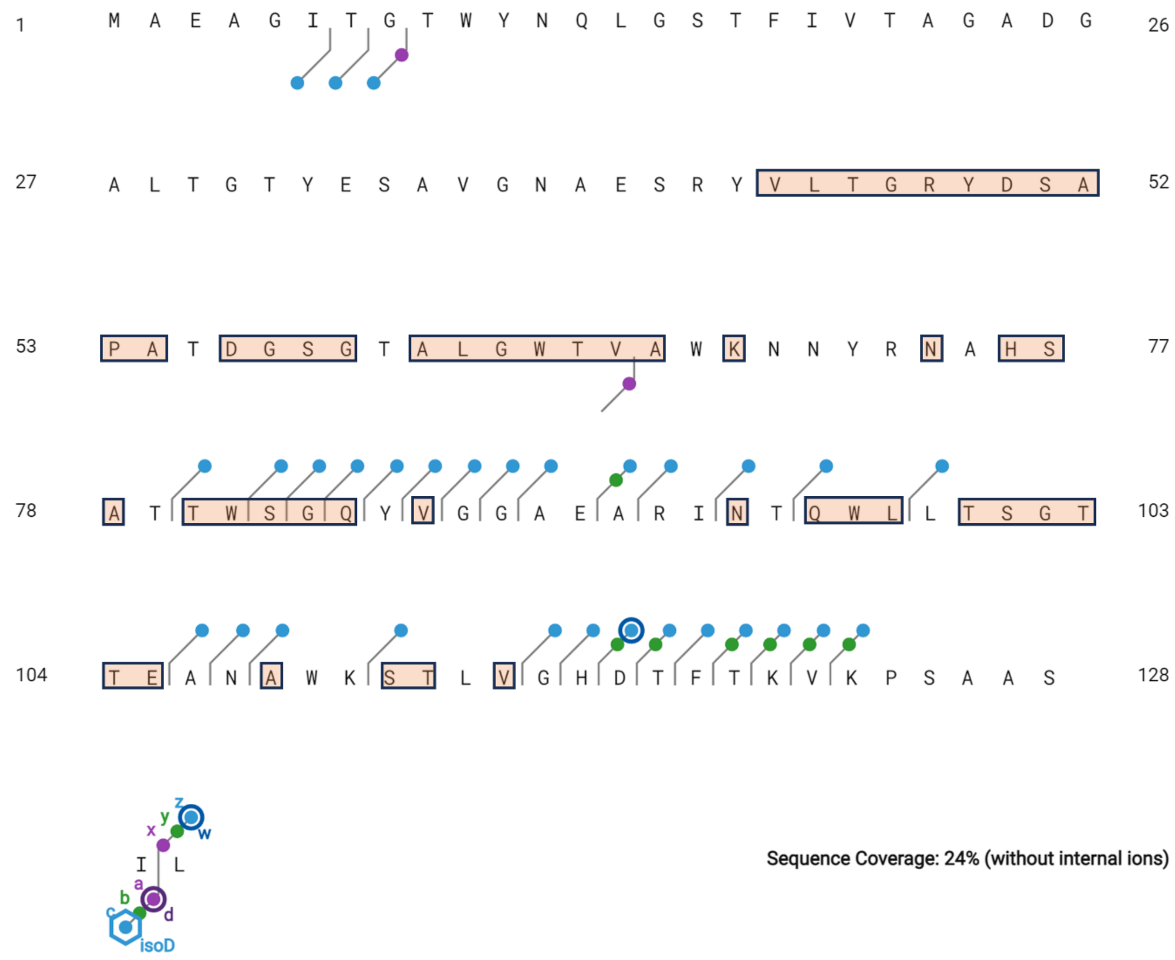

Figure S9: ECD sequence coverage map for SA 11+ after isCA with a cone voltage of 20 V and a Transfer CE of 100 V. The tan highlighted regions indicate interfacial residues identified by PISA interface analysis. These interfacial regions are comprised of an anti-parallel β-sheet. The residues between the highlighted sections are the hairpin loops.

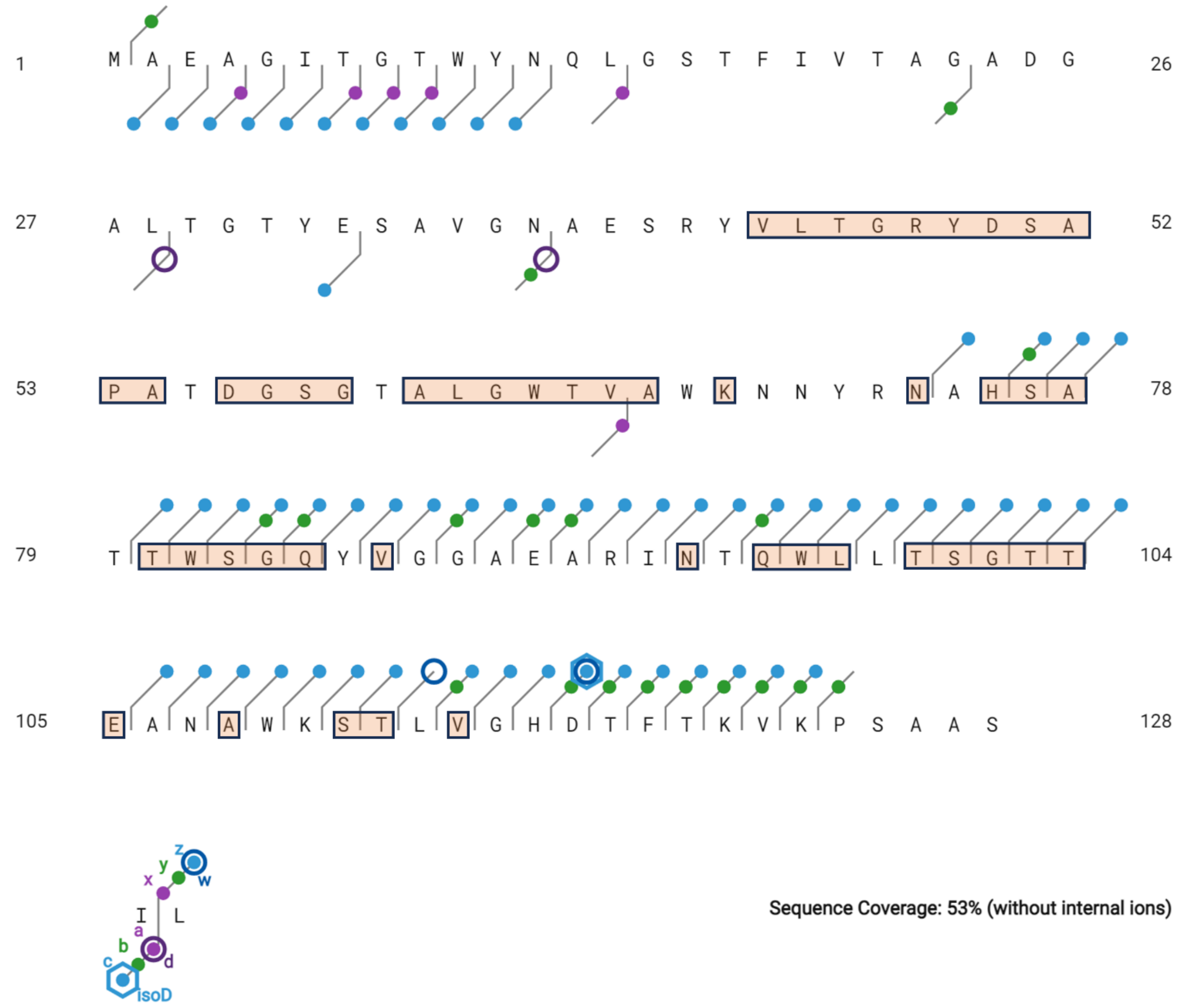

Figure S10: ECD sequence coverage map for SA 11+ after isCA with a cone voltage of 160 V and a Transfer CE of 100 V. The tan highlighted regions indicate interfacial residues identified by PISA interface analysis. These interfacial regions are comprised of an anti-parallel β-sheet. The residues between the highlighted sections are the hairpin loops. Compared to Figure S9, ECD after isCA with a cone voltage of 160 V clearly resulted in greater sequence coverage, which seems to be present along the interfacial area. This suggests that isCA rearranged the interfaces between SA subunits, allowing this region to become more exposed.

References

(1) Harvey, S. R.; Seffernick, J. T.; Quintyn, R. S.; Song, Y.; Ju, Y.; Yan, J.; Sahasrabuddhe, A. N.; Norris, A.; Zhou, M.; Behrman, E. J.; Lindert, S.; Wysocki, V. H. Relative Interfacial Cleavage Energetics of Protein Complexes Revealed by Surface Collisions. *Proc. Natl. Acad. Sci.* **2019**, *116* (17), 8143–8148. https://doi.org/10.1073/pnas.1817632116.
